# Trimester-Specific Reference Ranges for Thyroid Hormones in Iranian Pregnant Women

**DOI:** 10.1155/2013/651517

**Published:** 2013-06-09

**Authors:** Ladan Mehran, Atieh Amouzegar, Hossein Delshad, Sahar Askari, Mehdi Hedayati, Golshan Amirshekari, Fereidoun Azizi

**Affiliations:** ^1^Endocrine Research Center, Research Institute for Endocrine Sciences, Shahid Beheshti University of Medical Sciences, Tehran 1985717413, Iran; ^2^Mollecular and Cellular Endocrine Research Center, Research Institute for Endocrine Sciences, Shahid Beheshti University of Medical Sciences, Tehran 1985717413, Iran

## Abstract

*Background*. Due to many physiological changes during pregnancy, interpretation of thyroid function tests needs trimester-specific reference intervals for a specific population. There is no normative data documented for thyroid hormones on healthy pregnant women in Iran. The present survey was conducted to determine trimester-specific reference ranges for serum TSH, thyroxine (TT4), and triiodothyronine (TT3). *Methods*. The serum of 215 cases was analyzed for measurement of thyroid function tests by immunoassay method of which 152 iodine-sufficient pregnant women without thyroid autoantibodies and history of thyroid disorder or goiter were selected for final analysis. Reference intervals were defined as 5th and 95th percentiles. *Results*. Reference intervals in the first, second, and third trimesters were as follows: TSH (0.2–3.9, 0.5–4.1, and 0.6–4.1 mIU/l), TT4 (8.2–18.5, 10.1–20.6, and 9–19.4 *μ*g/dl), and TT3 (137.8–278.3, 154.8–327.6, and 137–323.6 ng/dl), respectively. No correlation was found between TSH and TT4 or TT3. Significant correlation was found between TT4 and TT3 in all trimesters (*r* = 0.35, *P* < 0.001). *Conclusion*. The reference intervals of thyroid function tests in pregnant women differ among trimesters. Applying trimester-specific reference ranges of thyroid hormones is warranted in order to avoid misclassification of thyroid dysfunction during pregnancy.

## 1. Background

Thyroid dysfunction, in particular hypothyroidism, can affect the health of both mother and fetus during pregnancy [1, 2]. Thyroid disorders are commonly present in pregnancy and puerperium. Hypothyroidism has a higher prevalence than hyperthyroidism (2.5 versus 0.2%) during the gestational period [3]. Early and appropriate detection of thyroid dysfunction and timely interventions improve maternal-fetal prognosis, so application of reliable gestational specific reference values for determining thyroid disorders in pregnant women would be a necessity [4].

The vast physiological changes in maternal hormones and their binding proteins complicate the assessment of normal levels of most hormones and the interpretation of the tests' result during gestation, especially when there are no established gestation specific reference intervals, as with Iranian population. Increase in thyroid size and production of thyroid hormones along with a 50% increase in the daily iodine requirement may result in hypothyroidism or unveil subclinical thyroid dysfunction in the later stages of pregnancy [5]. 

Despite the recent development in sensitive biochemical assays and understanding gestation-dependent trends of thyroid hormones, clinically useful gestation specific reference ranges are scarce [6]. Existing results are inconsistent and should not be extrapolated due to differences in ethnicity, maternal iodine status, laboratory assay method, and rigor for selection of reference population (choice of reference population, sample size, assessment of outliers, and so forth). There is also a doubt about the validity of the results as data are mostly derived from earlier studies [7], cross-sectional studies restricted to one trimester [8–11], use of less accurate hormone assays techniques, studies on population with differing iodine status [12, 13], applying outdated statistical methods for calculating reference ranges, and cross-sectional studies on different women from different gestations instead of longitudinal self-sequential surveys [12, 14]. Estimating self-sequential reference intervals [14] has narrower variations than interindividual variations caused by sampling error in cross-sectional studies conducted on different groups and hence provides clinicians with more accurate values for proper diagnoses and management.

Considering the lack of data regarding thyroid hormones specific reference ranges in Iranian pregnant women, we carried out this longitudinal study to investigate the self-sequential longitudinal reference values for thyrotropin (TSH), total thyroxine (TT4), and total triiodothyronine (TT3), in normal Tehranian pregnant women. 

## 2. Methods

### 2.1. Study Population

**Figure 1:**
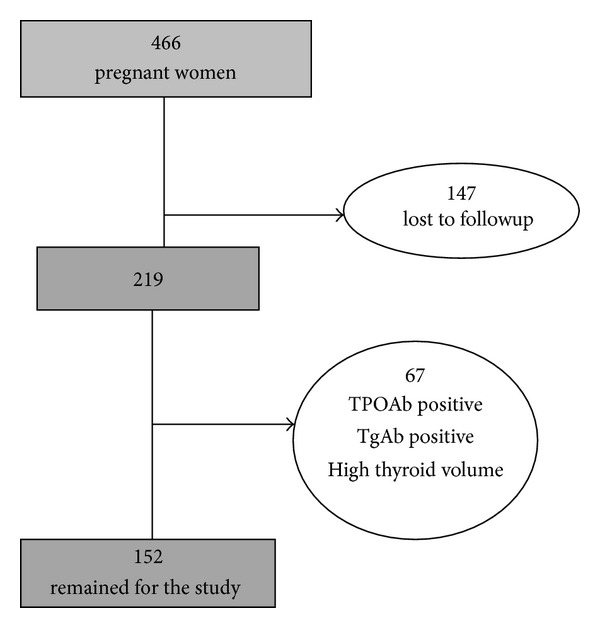


A total of 466 Iranian pregnant women, who had the same ethnicity of Persians, in the first trimester of pregnancy attending antenatal care clinics in the mother and child health care centers of two general hospitals of Tehran were consecutively recruited from November 2004 to November 2006. Only women with singleton pregnancies were enrolled. Inclusion criteria required documentation that thyroid-related measurements were available in all of the three trimesters. Of 466 women who were referred in the first trimester, 147 subjects were excluded because of preexisting thyroid disorders or nodules; those taking medications affecting thyroid function and those not available in all trimesters or lost to follow-up (referring elsewhere for delivery, nonviable pregnancy) were excluded, and 219 healthy pregnant women were selected. A further 67 subjects were excluded due to laboratory results of positive serum thyroid peroxidase and thyroglobulin antibodies (TPOAb > 40 IU/mL or Tg Ab > 100 IU/mL) (47 subjects), low urinary iodine level (<150 *μ*g/dL in two out of 3 sample measurements in the first trimester) (34 subjects), and enlarged thyroid gland (thyroid volume greater than 30 mL) by ultrasonography (9 subjects). None had overt hypo- (TSH > 4.5 mIU/L and T4 < 5.5) or hyperthyroidism (TSH < 0.1 mIU/L & T4 > 14.5) or subclinical hypothyroidism (TSH > 10 mIU/L). Those with subclinical hyperthyroidism (serum TSH levels under 0.1 mIU/L) were not excluded due to normal TSH suppression at pregnancy. Finally 152 healthy iodinesufficient women with viable, singleton pregnancies comprised the cohort study (Figure 1). 

### 2.2. Methods

Trained midwives informed participants about the rationale of the study to obtain written consent to allow for laboratory measurements and thyroid ultrasonography. Obstetric history was taken using a standard questionnaire, and physical examination was performed. Gestational age was calculated from the first day of the last normal menstrual period, and gestational age <14, 14–27, and >28 weeks comprised the first, second, and third trimesters of pregnancy. Serum samples were taken in all three trimesters for assessment of TSH, TT4, and TT3, thyroperoxidase antibody (TPOAb), and thyroglobulin antibody (Tg Ab). At initial presentation, before the end of the first trimester, three urine samples and in each of the second and third trimesters one urine sample were obtained for measurement of urinary iodine concentration (UIC). In all of the trimesters thyroid volume of pregnant women was also measured using ultrasound (Japan, Aloka, Portable 7.5 MHZ, SSD 2100 DX). The volume of each lobe was calculated by the formula *V* (mL) = 0.000479 × length × width × thickness (mm).

### 2.3. Laboratory Measurements

Urinary iodine concentration was measured in random urine samples using a manual method, based on the Sandell-kolthoff technique [25]. Measurement of TT4 and TT3 were done using the radioimmunoassay (RIA) method, and TSH was measured by immunoenzymometric assay (IRMA) using commercial kits (Izotop, Budapest, Hungray) and gamma counters (Wallac Wizard, Wallac Oy, Turku, Finland). Intra- and interassay coefficients of variations (CV) were 3.3 and 6.2% for TT4, 6.7 and 7.8% for TT3, and 3.9 and 7.1% for TSH, respectively. 

### 2.4. Statistical Analysis

 Since serum TSH had a non-Gaussian distribution, log-transformed values were used for TSH. The reference interval of each hormone in each trimester was determined by calculating the 5th and 95th percentiles (i.e., a central 90% interval) using the bootstrap technique. Repeated measures were employed to compare differences in thyroid hormones among groups with different gestational ages. Pearson's correlation was applied to evaluate the correlation of log-transformed TSH values with TT4 and TT3. All *P* values below 0.05 were considered statistically significant.

## 3. Results

Mean age of the whole study population (216 pregnant women) was 25.3 ± 5 years (range of 18–45). Thirty subjects (13.9%) were TPO Ab positive, 34 (15.4%) were Tg Ab positive, and 17 (7.9%) were positive for both TPO Ab and Tg Ab. After applying exclusion criteria, 152 pregnant women remained and entered the study cohort. Mean age of the cohort was 24.8 ± 4.9 years, of which 22 subjects (14.6%) were aged under 20 years, 106 (69.5%) between 20 and 30 years, and 24 (15.9%) over 30 years. The trimester average values of gestational age, urinary iodine, and thyroid volume of the study cohort are given in Table 1. Mean ± SD (range) gestational age at the study visits during the first, second, and third trimesters were 12.2 ± 3.7, 23.8 ± 1.8, and 35.7 ± 1.5 weeks, respectively.

Table 2 shows trimester specific percentiles (5th, 50th, and 95th) for TSH, TT4, and TT3 based on the data of this study. The limits of the reference intervals were calculated as *p* ^5^–*p* ^95^. Significant difference was observed in average values of TSH among trimesters (*P* = 0.008). TSH mean values rose significantly in the second trimester (*P* = 0.007) followed by a nonsignificant decrease in the third trimester (*P* = 0.8). The TSH reference intervals showed that lower TSH reference limit of 0.2 occurred in the first trimester in comparison with the 2nd and 3rd trimesters of 0.5 and 0.6, respectively. The upper reference range of TSH of 3.9 in the first trimester increased to 4.1 in the second trimester and remained unchanged in the last trimester.

The percentiles and average values of both TT4 and TT3 increased markedly after the first trimester reached a peak in the second trimester (*P* < 0.001) and declined in the third (*P* = 0.01 for TT4 and *P* = 0.5 for TT3) (Table 2). Significant difference was found in values of TT4 (*P* = 0.05) and TT3 (*P* < 0.001) between the first and the third trimesters.

**Figure 2:**
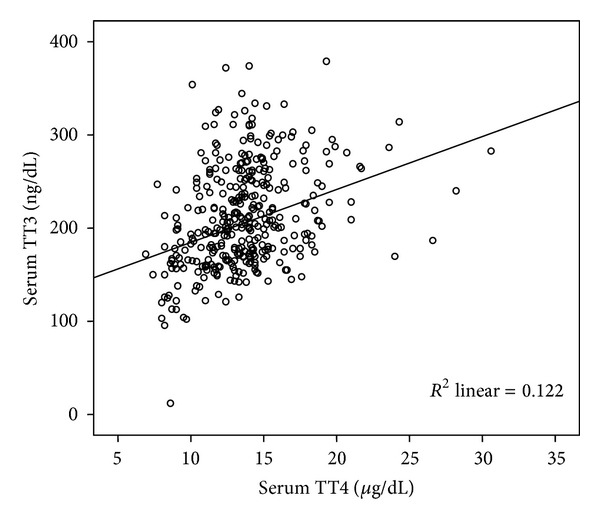
Scatter plot of correlation between TT4 and TT3 (*r* = 0.35,  *P* < 0.001).

Serum TSH had no significant correlation with TT4 and TT3. Significant correlations were found between TT4 and TT3 in all trimesters (*r* = 0.35, *P* < 0.001) (Figure 2). Considering each trimester separately, the correlation was stronger in the first trimester (*r* = 0.5, *P* < 0.001) compared with second and third trimesters (both, *r* = 0.26, *P* = 0.004).

## 4. Discussion

The present survey provides trimester-specific reference ranges for serum TSH and thyroid hormones during pregnancy in Tehran, I.R. Iran. Differences observed in the derived reference intervals between different gestations and in comparison with those provided by manufacturers in nonpregnant adults, and reports from the other countries provide further evidence of the importance of applying gestational age-specific reference value for a specific population in order to avoid misclassification of patients with thyroid dysfunction during pregnancy.

**Figure 3:**
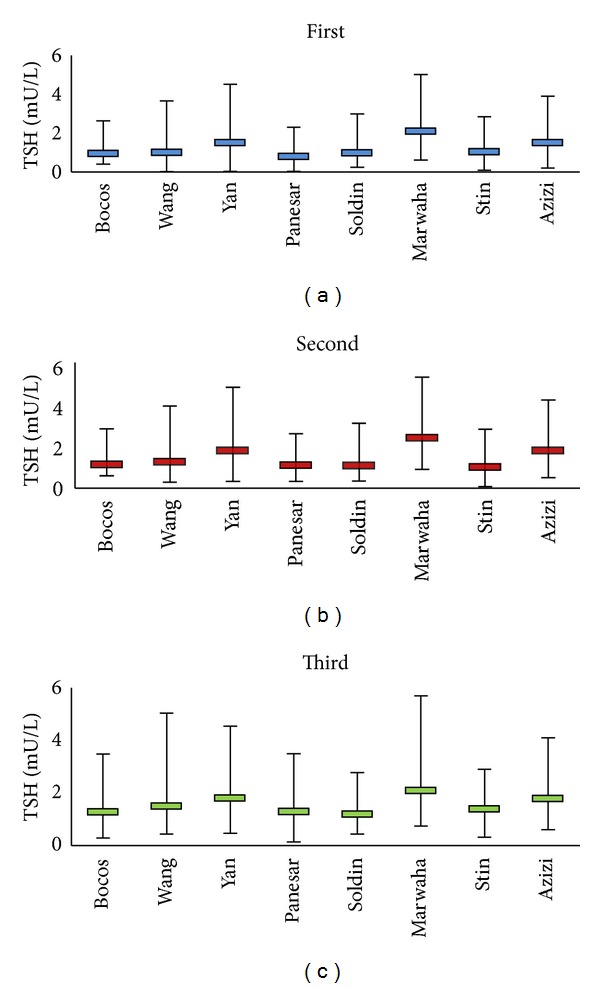
Comparison of trimester specific TSH reference ranges reported worldwide.

The major discrepancy in TSH reference values in our data compared with other reports mainly exists in the TSH upper reference limit, which is relatively higher than most of the other reports. However studies by Marwaha et al. in India [16] and Dhatt et al. in the United Arab Emirates [21] reported much higher TSH upper reference values than the present survey. Our TSH reference limits approximate those of Yu et al. from China [14], who used the same self-sequential design; we, however, used the 5th and 95th percentiles as the reference limits instead of the 2.5th and 97.5th used in their study. In agreement with previous studies our results indicate that the derived reference intervals of TSH for pregnant women were different (narrower and lower) from those proposed by kit manufacturers (0.2–4.5 mIU/L). Had we considered reference range of nonpregnant adults for a pregnant population, women with subclinical hypothyroidism would have been missed and classified in the normal group. For establishment of thyroid hormone reference intervals, many factors such as ethnic background, maternal iodine status, laboratory measurement techniques, definition of reference population, exclusion criteria, and method of statistical analysis need to be considered, which may justify the inconsistency in the results of different surveys.  Table 3 summarizes and compares (Figure 3) the reports of worldwide studies performed to date regarding trimester-specific reference intervals of TSH in pregnant women [15, 17, 18, 20, 22–24]. The studies were compared considering nationality, sample size and study design and method, although it would be more useful to make comparisons with specific factors in mind: for example, comparisons with other studies of women of similar ethnic *origin which is* more useful than country of origin *or *studies using the same assay platform, and so forth. 

Trimester-wise reference intervals for TT3 and TT4 were determined only in two studies by Yan et al. from China [18] and Soldin et al. [26]; both reported lower values for these hormones compared with our results. Trimester specific ranges in the first, second, and third trimesters in the study by Soldin et al. were 6.3–14.6, 6.4–14.8, and 6.3–16.7 *μ*g/dL for TT4 and 92–218, 112–278, and 111–265 ng/dL for TT3, respectively, using immunoassay method. In this study higher levels for TT4 and lower levels for TT3 in each case were reported using isotope dilution tandem mass spectrometry (LC/MS/MS). There was a significant correlation in TT4 measurements between IA and LC methods in all trimesters; in contrast to T3, the measurements of which were weakly correlated between the two methods. While TT4 was not significantly different during any trimester, TT3 increased significantly with the progression of pregnancy. T3 and T4 tended to be associated in all trimesters except for the third trimester. In the Chinese study by Yan et al. TT4 and TT3 were markedly increased at the first trimester, peaked in the second trimester, and declined slightly in the third trimester [18]. 

The strengths of our study include the strict inclusion criteria used as we considered maternal iodine status by measurement of urinary iodine level, thyroid size by ultrasonography and positivity for both TPO Ab and Tgb A, factors which have not been considered together in most studies. The exclusion criteria in the present study is specific and derived from the combination of recommendations by National Academy of Clinical Biochemistry (NACB) [27] and the National Health and National Examination Survey (NHANES) [28] to provide a well-defined healthy population. In addition, maternal iodine sufficiency in our survey is verified by urinary iodine measurement and not by just the assumption of iodine sufficiency in a specific area as done in most studies. Another strength of much importance is the self-sequential longitudinal design of our study which reduces the variation in reference intervals by omitting interindividual variations and reflects the changes of thyroid hormones during pregnancy more realistically than cross-sectional studies from different women in different stages. Additional strengths of our study are the adequacy of the sample size required for estimating reference values based on clinical laboratory standards (NCCLS) [29] and relevant application of nonparametric or parametric analyses based on the distribution of variables in our data, an important issue which has been ignored in some studies.

Our study does have some weaknesses. We did not have access to information on gestational and prenatal complications like hyperemesis, preeclampsia, gestational diabetes, premature delivery and fetal death, or anomalies and therefore did not consider them in the exclusion criteria. Gestational ages were based on the last menstrual period and not confirmed by ultrasonography. The current survey is limited by lack of data regarding the pre- and postpregnancy period although it does not interfere with the main goal of estimating trimester-wise reference value in our survey. It would have been better to have a sample of nonpregnant women simultaneously which would enable us to compare the changes occurring in these hormones more accurately.

## 5. Conclusion

The reference intervals of thyrotropin and thyroid hormones found in the current study differ from those reported by other countries and necessitate the importance of applying trimester-specific reference ranges specific to each population. Considering that Tehran is an area of iodine sufficiency and that women with urinary iodine excretion below 150 *μ*g/dL were excluded from the study, our results can be generalized to more than one million Iranian pregnant women each year in order to accurately detect thyroid dysfunction during pregnancy.

## Figures and Tables

**Table 1:** Average trimester specific values of gestational age, urinary iodine concentration and thyroid volumes in 152 healthy pregnant women.

Variable	1st trimester	2nd trimester	3rd trimester
Gestational time (week)	12.2 ± 3.7*	23.8 ± 1.8	35.7 ± 1.5
Urinary iodine (*μ*g/dL)	228 (169, 285)^†^	166 (108, 267)	143 (85, 232)
Thyroid volume (mL)	9.05 ± 4.1*	10.15 ± 4.3	10.9 ± 5.1

*Mean ± SD, ^†^median values (inter quartile range).

**Table 2:** Gestation specific percentile values for TSH, TT4, and TT3 in a self sequential cohort of 152 pregnant women with normal singleton pregnancies.

	Observed centiles		
	5th	50th	95th
TSH (mU/L)			
First trimester	0.2	1.5	3.9
Second trimester	0.5	1.8	4.1
Third trimester	0.6	1.8	4.1
TT4 (*µ*g/dL)			
First trimester	8.2	12.9	18.5
Second trimester	10.1	14	20.6
Third trimester	9.0	13.4	19.4
TT3 (ng/dL)			
First trimester	138	190	278
Second trimester	155	221	328
Third trimester	137	228	324

**Table 3:** Summary of worldwide studies reporting trimester-specific reference intervals for TSH during pregnancy.

Study	Country	Sample size and Design	Centiles' used	TSH reference intervals		
				1st trimester	2nd trimester	3rd trimester
Panesar et al. [12], 2001	China	343 cohort	2.5th, 97.5th	*n* = 158 0.03–2.3	*n* = 117 0.03–3.1	*n* = 76 0.13–3.5
Soldin et al. [6], 2007	USA	261 cross-sectional	2.5th, 97.5th	*n* = 71 0.24–2.99	*n* = 83 0.46–2.95	*n* = 62 0.43–2.78
Stricker et al. [15], 2007	Switzerland	2272 cross-sectional	2.5th, 97.5th	*n* = 783 0.09–2.82	*n* = 528 0.2–2.79	*n* = 501 0.31–2.9
Marwaha et al. [16], 2008	India	541 cross-sectional	5th, 95th	*n* = 107 0.6–5	*n* = 137 0.43–5.78	*n* = 87 0.74–5.7
Bocos-Terraz et al. [17], 2009	Spain	1198 cross-sectional	2.5th, 97.5th	*n* = 481 0.03–2.65	*n* = 243 0.12–2.64	*n* = 297 0.23–3.56
Yu et al. [14], 2010	China	538 cohort	2.5th, 97.5th	*n* = 301 0.02–3.65	*n* = 301 0.36–3.46	*n* = 301 0.44–5.04
Yan et al. [18], 2011	China	505 cross-sectional	2.5th, 97.5th	*n* = 168 0.03–4.51	*n* = 168 0.05–4.5	*n* = 169 0.47–4.54
Azizi et. al, 2012 [19]	Iran	261 cohort	5th, 95th	*n* = 216 0.2–3.9	*n* = 216 0.5–4.1	*n* = 216 0.6–4.1
Haddow et al. [20], 2004	USA	1126 cohort	5th, 95th	*n* = 1005 0.08–2.73	*n* = 1005 0.39–2.70	—
Dhatt et al. [21], 2006	United Arab Emirates	1140 cross-sectional	—	United Arabs 0.06–8.3, *n* = 97 other Arabs 0.04–9.3, *n* = 122 Asians 0.12–7.4, *n* = 79	United Arabs 0.17–5.9 other Arabs 0.23–5.7 Asians 0.3–5.5	—
Lambert-Messerlian et al. [22], 2008	USA	9562 cohort	98th, 2th	*n* = 9562 0.13–4.15	*n* = 9562 0.36–3.77	—
Santiago et al. [23], 2011	Spain	429 cross-sectional	97th, 3th	*n* = 279 0.23–4.18	*n* = 210 0.36–3.89	—
Karakosta et al. [24], 2011	Greece	425 cohort	2.5th, 97.5th	*n* = 143 0.05–2.53	*n* = 260 0.18–2.73	—
